# Ethnopharmacological Survey of Medicinal Plants Used by Traditional Healers and Indigenous People in Chittagong Hill Tracts, Bangladesh, for the Treatment of Snakebite

**DOI:** 10.1155/2015/871675

**Published:** 2015-03-23

**Authors:** Mohammad Fahim Kadir, James Regun Karmoker, Md. Rashedul Alam, Syeda Rawnak Jahan, Sami Mahbub, M. M. K. Mia

**Affiliations:** ^1^Department of Pharmaceutical Technology, Faculty of Pharmacy, University of Dhaka, Dhaka 1000, Bangladesh; ^2^Department of Pharmacy, University of Asia Pacific, Dhaka 1209, Bangladesh; ^3^Bangladesh National Herbarium, Bangladesh

## Abstract

Snakebites are common in tropical countries like Bangladesh where most snakebite victims dwell in rural areas. Among the management options after snakebite in Bangladesh, snake charmers (*Ozha* in Bengali language) are the first contact following a snakebite for more than 80% of the victims and they are treated mostly with the help of some medicinal plants. Our aim of the study is to compile plants used for the treatment of snakebite occurrence in Bangladesh. The field survey was carried out in a period of almost 3 years. Fieldwork was undertaken in Chittagong Hill Tracts, Bangladesh, including Chittagong, Rangamati, Bandarban, and Khagrachari. Open-ended and semistructured questionnaire was used to interview a total of 110 people including traditional healers and local people. A total of 116 plant species of 48 families were listed. Leaves were the most cited plant part used against snake venom. Most of the reported species were herb in nature and paste mostly used externally is the mode of preparation. The survey represents the preliminary information of certain medicinal plants having neutralizing effects against snake venoms, though further phytochemical investigation, validation, and clinical trials should be conducted before using these plants as an alternative to popular antivenom.

## 1. Introduction

Snakebite, caused by a bite from a snake, is an accidental injury, which results in puncture wounds inflicted by the animal's fangs and sometimes causes envenomation. Snakes are carnivorous vertebrates of the class Reptilia, order Squamata, and suborder Serpentes. Snakes usually kill their prey with constriction rather than venom, though venomous snakes can be found on every continent except Antarctica [1]. 15% of the almost 3000 known species of snakes are venomous [2–4] and, in South Asia, four species were thought to be responsible for causing almost all the deadly cases of venomous snakebites which are referred to as the “big four,” which include the Indian cobra (*Naja naja*), the common krait (*Bungarus caeruleus*), Russell's viper (*Daboia russelii*), and the saw-scaled or carpet viper (*Echis carinatus*). However, other venomous snakes may also be found in this area [5–7] and thus represent a major cause of morbidity and mortality to humans [8–11]. Exact numbers on the global prevalence of snakebites and the percentage of severe or fatal cases are largely unknown [12]. However, at least 421,000–1,841,000 envenoming and 20,000–94,000 deaths occur worldwide each year due to snakebite [1]. According to Williams et al. [13] these events surpass the number of deaths from tropical diseases such as hemorrhagic fever, dengue, cholera, leishmaniasis, and the Chagas disease.

Incidence of snakebites in Bangladesh is very high like other tropical countries of Southeast Asia [1]. Here most snakebite victims dwelling in rural areas are farmers, fishermen, and hunters [7–9] and also there are a high number of snakebite occurrences that happened at their homes as most of the snakes are nocturnal animals and poor people have the practice of sleeping on the floor [7]. An epidemiological study estimated about 8000 snakebites per year with 22% mortality which has been identified to be one of the highest in the world [10, 14]. Nonetheless, there are approximately 80 species of snakes found in Bangladesh; among them only few are venomous. These are cobra, krait, Russell's viper, saw-scaled viper, green snakes, and sea snakes. However, most of the bites are reported by nonvenomous snakes and even as many as 40% bites inflicted by venomous snakes do not produce signs of envenoming [15].

Antivenom is the only therapeutic agent against snake venom available throughout the world. These antivenoms have highly effective neutralizing systemic effects but show some limitations in the inhibition of the local disorders [16, 17] and also a chief drawback of serum therapy is its excessive cost and likelihood that victims are often at some distance away from availability of modern treatment when bitten as antivenom treatment should be sought as soon as possible for their potential efficacy. Moreover, there is a crisis in the quality and supply of antivenom serum in the rural areas where most incidences of snakebites occurred [18]. These problems could be subsided by using traditional plant based treatment since approximately 700 plant species are known to possess potential antivenom [19–22].

Ethnopharmacological survey is important for the conservation and utilization of biological resources [23] since of the 422,000 flowering plants found globally [24] more than 50,000 are used for medicinal purposes [25] and these plants contribute to 33% of drugs produced worldwide [26]. To date approximately more than 6,000 species of indigenous and naturalized plants have been identified out of which more than one thousand contain medicinally useful chemical substances [27, 28]. Due to this rich diversity apparently more than 80% of the Bangladeshi use alternative (Ayurveda, Siddha, Unani, and Homeopathy) medicines for their healthcare and herbs constitute a major source of these alternative systems of medicine [29, 30].

Several ethnobotanical investigations have been carried out at different parts of the world to explore the herbal treatment against snakebite [16, 31–35]. But there are very few ethnobotanical surveys carried out in Bangladesh to explore the medicinal plants used here in the treatment of snakebite. The present study was conducted in order to document the traditional knowledge of the medicinal plants used by the traditional healers of Bangladesh for treating against snakebite.

## 2. Materials and Methods

### 2.1. Study Area

**Figure 1:**
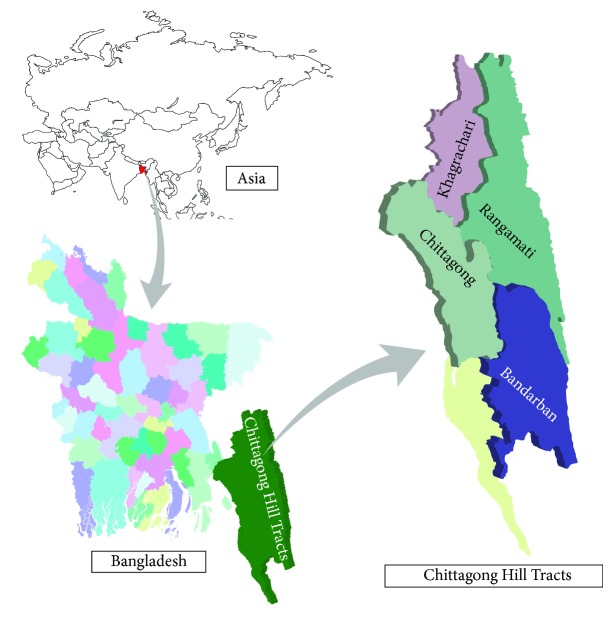
Map of Chittagong Hill Tracts, Bangladesh.

The study was conducted in four districts in Chittagong Hill Tracts (Figure 1) in Bangladesh which is located in South Asia and bordered by India and Myanmar and by the Bay of Bengal to the south (latitudes 20° and 27°N and longitudes 88° and 93°E) with population over 162 million having 35 smaller groups of indigenous people. The vegetation type of the study area falls under tropical evergreen and semievergreen forests. More than 3 million people live in this study area and these people mostly depend on the resources coming from the hilly areas [36].

### 2.2. Informants and Ethnomedicinal Data Collection

The survey was conducted in the official language of Bangladesh, Bengali language, from January 2010 to December 2012. Objectives of the survey were explained to the local communities during social gatherings arranged by local people familiar with well-known traditional health practitioners (THPs). While meeting with indigenous populations who had mother language different from the state language, help from local bilingual translator was taken. Special emphasis was given in seeking out people who had the empirical knowledge on medicinal plants and experience in the use of traditional medicinal plants. Personally administered method was followed during the survey. Open-ended and semistructured questionnaire was used [37, 38] for this survey seeking for the following information: (a) the local name, (b) plants part/s used, (c) the method of preparation, (d) solvent/adjuvant used, (e) mode of application, (f) gastrointestinal and other medicinal uses, (g) voucher specimen number, and (h) dose and dosage forms. After completion of survey, consultation with Botanist Mr. Manzur-ul-Kadir Mia, M.D., Former Principal Scientific Officer and Consultant of Bangladesh National Herbarium, Dhaka, was carried out for getting identification, scientific names, family names, habit, habitat, nature, relative abundance, and preservation of the species. The voucher specimens of the plants were deposited in Bangladesh National Herbarium, Dhaka (DACB).

### 2.3. Data Analysis

All the species were listed in alphabetical order by their scientific name, family, local name, general name, plants parts used, mode of preparation, habit, habitat, relative abundance, nature, general name, solvent used, and frequency of citation (FC). Here FC is defined as the ratio of “number of times a particular species was mentioned” and “total number of times that all species were mentioned” multiplied by 100. All the data such as frequency distributions were calculated by using SPSS 16.0.

## 3. Results

### 3.1. Informants

Among the 110 interviewees, major informants were male (65%), aged (regardless of gender) 50–60 years (31%), mostly with 5 years of institutional education (44%), and having 10–20 years of relevant experience (34%) (Table 1).

### 3.2. Plants Using in Treatment of Snakebite and Other Relevant Information

**Figure 2:**
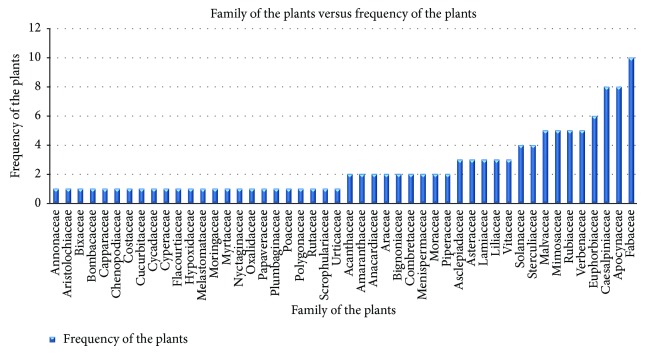
Family of the plants with their frequencies.

116 plant species belonging to 48 plant families have been identified as being used in the treatment of snakebite by traditional healers in Bangladesh. The largest number of species was noted from the family Fabaceae (10 species), followed by Apocynaceae (8 species), Caesalpiniaceae (7 species), and Euphorbiaceae (6 species) (Figure 2).

**Figure 3:**
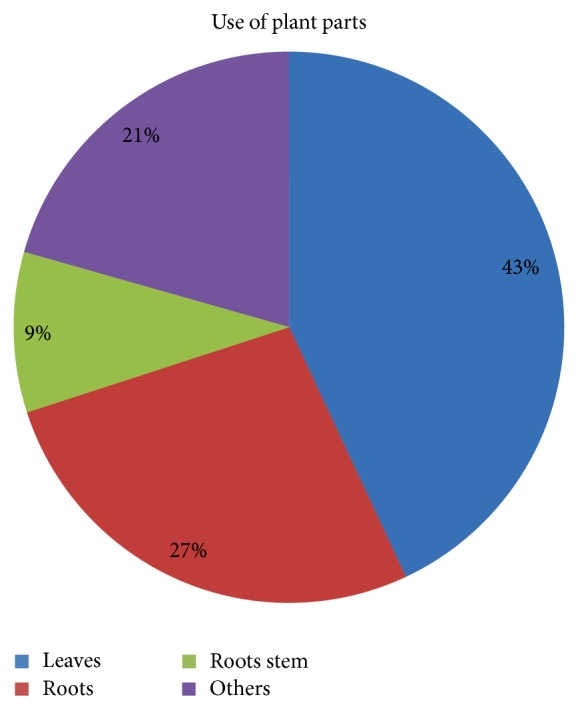
Percentage of plant parts used.

**Figure 4:**
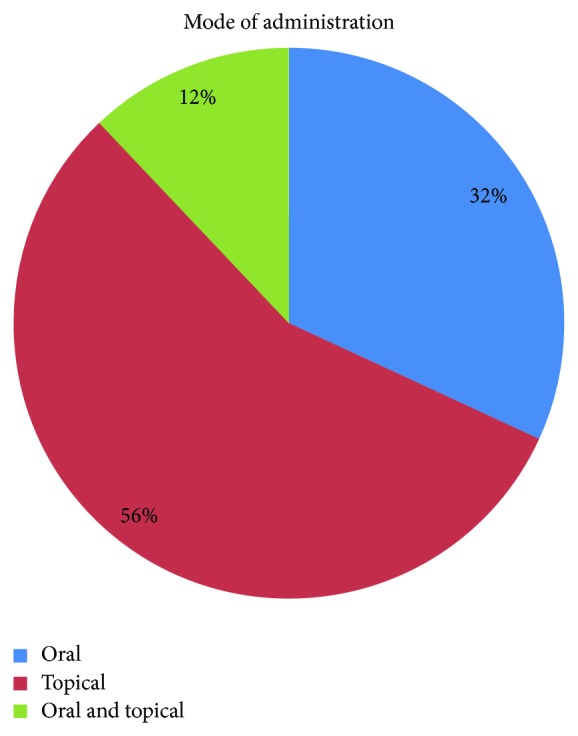
Percentage of mode of administration.

Leaves (43%) were the most frequently used plant parts, followed by roots (27%) and roots stem (9.4%) (Figure 3). The major mode of preparation is paste (69.3%) followed by juice (21%) and powder (11.23%). Preparations were made with water, honey, wine, lime water, and milk as solvent. The mode of administration was oral (31.9%), topical (56.03%), and oral and topical (12.07%) (Figure 4). 32% of the reported species were herb which was followed by tree (23.3%) and climber (9.5%). Most of the plants are wild (70%) and some are cultivated (18%), whereas others are both cultivated and wild (Table 2). The species* Rauvolfia serpentina*,* Allium cepa*,* Aristolochia indica*,* Costus speciosus*,* Emblica officinalis*,* Hemidesmus indicus*,* Leucas aspera*, and* Vitex negundo *were the most frequently cited in study area. The doses of the available plants are presented in Table 3.

## 4. Discussion

Fabaceae is the most dominant family in the current investigation. This is perhaps because of worldwide prevalence of the species from this family [112, 113]. Leaves were the major plant parts used solely or mixed with other parts in the treatment of snakebite. Ease of collection of leaves is the prime reason compared to roots, flowers, and fruits [114–116]. On the other hand, herbs and trees were the most common habit of the reported plants which might be attributed to the huge number of trees or herbaceous plants naturally abundant in this hilly area [117].

It was very common that blend of different adjuvant including other plant parts was used for the preparation of medication to counteract snake venom. Several researchers also reported this kind of polyherbal treatment [118–121]. The frequent use of multiple plant remedies might be illustrated by the phenomenon of synergistic actions where two or more plants produce an effect greater than the sum of their individual effects [122]. This is particularly true in case of medicinal plant treatment, since each medicinal plant contains numerous pharmacologically active compounds [118].

Among the management options after snakebite, snake charmers (*Ozha* in Bengali language) were the first contact following a snakebite for more than 80% of the victims in these areas [10]. We also noticed that the field of “snakes and snakebite” has a mythological fragrance in the mind of people living here. The* Ozha* not only depends on herbal remedies but also recites* mantras* (magical/mystical words) to enthrall people. There are also potentially harmful approaches reported few of which are making multiple incisions around the bite site, incorrect application techniques in tourniquets (e.g., wrong pressure), and sucking blood orally from the multiple cuts which are practiced in an alarmingly high proportion of cases.

The species with high FC values is a sign of their diverse and numerous medicinal activities and thus it offers further pharmacological, toxicological, and phytochemical analysis for the discovery of potential novel drugs.

Snake venom contains a complex mixture of enzymes, nonenzymatic proteins, carbohydrates, lipids, and other substances [123–126] most of which are extremely toxic. Snakebite envenoming has cytotoxic, hypotensive, neurotoxic, or anticoagulant effects [127]. Cytotoxic enzymes, phospholipases A_2_ and metalloproteinases, activate proinflammatory mechanisms that result in edema, blister formation, and local tissue necrosis and facilitate the release of bradykinin, prostaglandin, cytokines, and sympathomimetic amines that cause the intense pain [128]. In addition, there are some venom toxins including aminopeptidases having the ability to alter the physiological function of the victims and ultimately causing systemic hypotension [126]. Many snake venoms have peptides that inhibit angiotensin-converting enzyme causing a slump in arterial blood pressure [129]. Moreover, some toxins such as safarotoxins and endothelins are potent vasoconstrictors of coronary arteries and might be responsible for myocardial ischemia or cardiac arrhythmias [123]. Neurotoxins cause paralysis by affecting the neuromuscular transmission at either presynaptic or postsynaptic levels [130]. Presynaptic neurotoxins, also called b-neurotoxins, include taipoxin, paradoxin, trimucrotoxin, viperotoxin,* Pseudocerastes*, textilotoxin, and crotoxin [127] which are phospholipase A_2_ complexes that inhibit the release of acetylcholine from the presynaptic terminal [131, 132]. On the other hand, postsynaptic neurotoxins including irditoxin [127] called a-neurotoxins cause a reversible blockage of acetylcholine receptors [133–135]. Snake venom toxins may also interfere with blood coagulation and cause hemorrhages or thrombosis [125, 127, 136, 137].

Elucidation of the mode of actions of 116 plants individually is beyond the scope of this study. Research suggests extract of different medicinal plants having antivenom activities such as reducing necrotic and hemorrhagic activity as well as preventing cardiac arrest and reversing the effect of paralysis of skeletal muscle caused by snake venom. Also they might inhibit phospholipase A_2_ that causes degranulation of mast cell [138] and consequently they prevent release of platelet activating factors and histamine into circulation, preventing hypersensitive anaphylactic reaction [139].

Several studies have been conveyed in finding of active constituents in the plants used against snake venom. Among the 116 plants in this study, the phytochemical investigations are conducted in most of the plants though the compounds rational for antivenom properties are still unknown for most of them. Extensive phytochemical investigations on the plants mentioned in this study could be another mammoth task. Several plant constituents like flavonoids, quinonoid, xanthene, polyphenols, terpenoids lupeol, gymnemagenin, and pentacyclic triterpenes like oleanolic acid, ursolic, tannins, taraxasterol, amyrin, and so forth are found to be present in varying proportions in surveyed plants. These compounds have also been previously tested in vitro for possessing protein binding and enzyme inhibiting properties [140–142].

These literature studies revealed that the alkaloids (*Eclipta prostrate*,* Rauvolfia serpentina*,* Strychnos nux-vomica*, and* Mimosa pudica*), esters (*Gloriosa superba*), phenolic fraction (*Hemidesmus indicus*), terpenoids (*Aristolochia indica*,* Andrographis paniculata*), and flavonoids fraction (*Tephrosia purpurea*) neutralized the snake venom activities. Flavonoids have been shown to inhibit phospholipases A_2_, an important component of snake venoms [143]. The antivenom effects of wedelolactone, a coumestan isolated from the* Eclipta prostrate*, are well cited for antivenom activities [144]. 2-Hydroxy-4-methoxy benzoic acid, found in* Hemidesmus indicus* root extracts, was identified as a snake venom neutralizing factor which effectively neutralized viper venom induced lethal, hemorrhagic, coagulant, anticoagulant, and inflammatory activity [145]. This compound seems to act through free radical formation system [146] and is one of the mechanisms of venom inhibition. Caffeic acid is present in* Strychnos nux-vomica*, and the monomeric caffeic acid is a proven antidote against snake venoms when given as oral and parenteral administration [147]. Marmin in* Aegle marmelos*, a monoterpenoid substituted fernolin [148], has been mentioned as a remedy against snakebite. Piperine from* Piper nigrum* inhibits the adhesion of neutrophils to endothelial monolayers. Also it possesses inhibitory activities on prostaglandin and leukotrienes and thus possesses anti-inflammatory activity [149–151]. Quercetin is a potent inhibitor of lipoxygenase, and free quercetin and its glycosides rutin are present in* Allium cepa* skins [152]. The aristolochic acid content of* Aristolochia indica* contains a large number of proteins that cluster under native condition. It shows strong gelatinolytic, collagenase, nuclease, and peroxidase activities. It interacts with the components of snake venom and partially inhibits proteolytic and L-amino acid oxidase activities of the venom [12]. Active principle of* Bauhinia forficata* has thrombin-like enzyme that acts as potent inhibitor of clotting activity that otherwise causes persistent hemorrhage [153].

Most of the plants documented in this study are used for the treatment of versatility of disease. This trend is a possible indication of the tradition of THPs to develop local healing system through trials and errors for optimal treatment practices [154].

There are resemblances in comparative studies of these cited plants to other surveys regarding medicinal plants having antivenin characteristics (Table 4). Using the same plants in different areas by different cultures for the same purpose might be considered as a justification of their pharmacological efficacy [155].

12 of these cited plants had been found to possess possible toxic potentiality (Table 5). However, among those possibly the most toxic one is* Abrus precatorius*. It contains abrin, a serious toxic compound, which after penetrating the cells of the body inhibits cell protein synthesis. Human fatal dose of abrin is approximately 0.1–1 mg/kg. But toxins are released only if the seed is chewed and swallowed [91]. Another dangerous plant is* Ageratum conyzoides* which in ingestion can cause liver lesions and tumors [94, 95]. There was a mass poisoning incident reported in Ethiopia as a result of contamination of grain with* A. conyzoides* [96]. In addition, epidemic dropsy and ocular toxicity have been reported by seed oil of* Argemone mexicana* [98–101] and latex of* Calotropis procera* [105], respectively; the rest are toxic only due to high doses of ingestion. However, a number of phytochemical investigations would be required to declare these plants as being toxic.

## 5. Conclusion

This survey represents the contribution of natural flora of Bangladesh to the global approach in the management of snakebite occurrences. The knowledge documented in this study possibly supports the development of novel plant based treatment. Further investigations should be carried on especially in order to ensure safe therapy concerning medicinal plants. Therefore, snake charmers should be trained on as a priority basis. Again, scarcity of supply of snake antivenin is a major factor which needs to be addressed by local production. And in that case these findings regarding herbal antidote would be useful in planning and formulating strategies and specific interventions to combat snakebite related health problems in Bangladesh.

## Figures and Tables

**Table 1:** Demographic data of the informants.

Variable	Categories	Frequency (*n* = 110)	Percentage
Gender	Male	72	65
	Female	38	35

Age (years)	<30	16	15
	30–40	25	23
	40–50	23	21
	50–60	34	31
	>60	12	11

Education (years)^†^	0^*^	20	18
	5	48	44
	8	21	19
	10	15	14
	12	12	11
	16	10	10
	>16	4	4

Experience^‡^	<2	18	16
	2–5	25	23
	5–10	18	16
	10–20	37	34
	>20	12	11

Profession^Ұ^	Traditional health practitioners	42	38
	Indigenous people	68	62

^*^These people do not have any formal educational training; ^†^year completed through formal educational institution; ^‡^relevant to treating people; ^Ұ^people who acquired medicinal knowledge by themselves and are usually involved in profession not relevant to medicine.

**Table 2:** Scientific names and other relevant information.

S/L number	Scientific name	Bangla/Bengali name	English name	Family	Habit	Habitat	Nature	Plants parts used	Preparation	Solvent/ adjuvant	Mode of application	FC	Voucher specimen
1	*Abelmoschus moschatus *Medic.	Latakasturi	Musk mallow	Malvaceae	Hf, Rs	W	Fr, L, S	J, Pa	M/sugar, W	O, T	Hf, Rs	0.25	MFK 240 (DACB)

2	Abroma augusta Linn. f.	Ulatkambal	Devil's cotton	Sterculiaceae	Sh	Hf	W	L, R, St	Pa	H, W	T	1.59	MFK 75 (DACB)

3	*Abrus precatorius *L.	Kunch, Rati	Indian liquorice	Fabaceae	Cl	Hf, Hs	W	S	P	*Andrographis paniculata*, lemon juice	O	0.12	JRK 111 (DACB)

4	*Acacia farnesiana* (L.) Willd., *Vachellia farnesiana* (L.) W. and A.	Belati babul, Gokul	Needle bush	Mimosaceae	T	G	Cu	R	Pa		T	0.13	JRK 127 (DACB)

5	*Acalypha indica* L.	Mukta jhuri	Indian nettle	Euphorbiaceae	H	Rs, Wp	Cu, W	Wp	Pa		T	0.21	MSBS 143 (DACB)

6	*Achyranthes aspera* L.	Apang	Prickly chaff flower	Amaranthaceae	Sh/Wh	Rs, Wp	W	L, S, Wp	P		O, T	2.37	JRK 172 (DACB)

7	*Acorus calamus* L.	Bach, safed bach, shet bach	Sweet flag	Araceae	H	Mp	Cu	Rh	Pa	W	O	0.15	JRK 97 (DACB)

8	*Aegle marmelos* (L.) Corr. Serr.	Bel, bela, bilbo	Bael fruit, Bengal quince	Rutaceae	T	Hs	Cu	Wp	Pa	Rice beer	O, T	2.41	JRK 37 (DACB)

9	*Ageratum conyzoides* L.	Sahadevi	Floss flower, goat weed, white weed	Asteraceae	H	Wp	W	L	Pa		T	0.14	JRK 43 (DACB)

10	*Albizia lebbeck* (L.) Benth.	Siris, Sirisha	Siris tree	Mimosaceae	T	F	Cu	Fl	Pa		T	0.18	MFK 209 (DACB)

11	*Albizia procera* (Roxb.) Benth.	Korai	Golden trumpet	Mimosaceae	T	Hf, Hs	Cu, W	R	Pa		T	0.22	JRK 17 (DACB)

12	*Allium cepa* L.	Palandu, Piyaj	Onion	Liliaceae	H	G	Cu	Bu	J	Mustard oil	O	3.87	JRK 35 (DACB)

13	*Alstonia scholaris *R. Br.	Chatim	Devil's Tree, Dita Bark Tree	Apocynaceae	T	Hf	W	B, Fl, G, L, R	D, J	M, W	O	2.32	MFK 110 (DACB)

14	*Amaranthus spinosus* L.	Kanta note	Amaranthus	Amaranthaceae	H	Hf, Rs, Vt	W	L, R, Wp	I, Pa	W	T	0.30	MSBS 43 (DACB)

15	*Andrographis paniculata *(Burm. f.) Wall ex Nees., *Justicia paniculata* Burm. f.	Kalmegh, Maha-tita	Creyat root	Acanthaceae	H	Vt	W	L, R	Pa	H, W	O	0.29	JRK 57 (DACB)

16	*Annona squamosa* L.	Ata, Sharifa	Custard apple of India, sweet or sugar apple of the W. Indies and America	Annonaceae	T	F	Cu	Fr	J	W	T	0.34	MFK 12 (DACB)

17	*Argemone mexicana* L.	Baro shial kanta, shial kanta	Mexican or prickly poppy	Papaveraceae	Sh	Hf	W	L, S	Pa		T	0.24	MFK 176 (DACB)

18	*Aristolochia indica* L.	Isharmul	Indian birthwort	Aristolochiaceae	Wh	F, Hf	W	L, R	Pa	H	O	3.53	MSBS 61 (DACB)

19	*Asparagus racemosus *Willd.	Satamuli	Chinese gooseberry	Liliaceae	Cl	Hf, Hs	Cu, W	L	Pa		T	2.50	MSBS 79 (DACB)

20	*Bacopa monnieri* (L.) Pennell	Brahmi sak, adha birni, Dhop chamni	Thyme-leaved gratiola	Scrophulariaceae	Cr	F, Hf	W	L	P	M/black tea	O	0.04	MFK 144 (DACB)

21	*Baliospermum montanum *L.	Danti mool	Wild croton	Euphorbiaceae	Sc	Hf	W	L	Pa		T	0.06	JRK 10 (DACB)

22	*Bauhinia variegata* L.	Rokto kanchan, sweet kanchan	Camel's foot tree, mountain tree, orchid tree	Fabaceae	T	Rs	Cu	B, St	Pa		T	0.11	JRK 28 (DACB)

23	*Begonia barbata *C.B. Clarke	Bini gach		Bignoniaceae	H	Mp	W	L, St	Pa		T	0.17	JRK 68 (DACB)

24	*Bixa orellana *L.	Utkana	Monkey turmeric	Bixaceae	T	Hs	Cu	Fr, L, R, S	D	W	O	1.85	JRK 91 (DACB)

25	*Bombax ceiba* L., *B. malabaricum *DC.,* Salmalia malabarica* Schott. & Endl.	Simul, Shimul, Rokto simul	Red cotton tree, silk cotton tree	Bombacaceae	T	Hf	Cu	Fr, S	Pa	H/black peppers seed, camphor	O	0.31	MSBS 45 (DACB)

26	*Buchanania lanzan* Spreng., *B. latifolia* Roxb.	Piyal, Chikki	Chironji tree	Anacardiaceae	Cl	F	W	B	Pa		T	0.10	MFK 14 (DACB)

27	*Butea monosperma* (Lamk.) Taub., *B. frondosa* Koen ex Roxb.	Palas	Butea gum tree, flame of the forest, parrot tree, Bastard teak, Bengal kino	Fabaceae	T	Rs	Cu	Stb	Pa	Z	O	0.18	MFK 65 (DACB)

28	*Byttneria pilosa *Roxb.	Harjora		Sterculiaceae	Sc	Hf	W	L, St	Pa		T	0.11	MSBS 92 (DACB)

29	*Cajanus cajan *(L.) Huth.	Arhar	Pigeon pea	Fabaceae	S	Hs	Cu	S	Pa	Leaf juice of *Senna tora *	T	1.81	MSBS 113 (DACB)

30	*Calotropis procera *(Ait.) Ait. f.	Choto akanda, sweet akanda	Swallow wort	Asclepiadaceae	Sh	Rs, Wp	W	Fl, La, R	J, P	Black pepper	O, T	0.47	JRK 56 (DACB)

31	*Calotropis gigantea* (L.) Ait. f.	Baro akanda, Gurtakand, sweet akand	Giant milk weed, swallow wort	Asclepiadaceae	Sh	Rs, Wp	W	R	Pa	M	O	0.55	MFK 169 (DACB)

32	*Calycopteris floribunda* Lamk.	Guichha Lata	*Calycopteris *	Combretaceae	Sc	Hf	W	R	J	W	T	1.55	MFK 173 (DACB)

33	*Capparis zeylanica* L.,* C. horrida *L. f.	Asahia, Baganai, Kalokera		Capparaceae	Sh	Vt	W	Fr, S	P	W	O	0.20	JRK 44 (DACB)

34	*Capsicum annuum* L.	Marich	Red pepper	Solanaceae	H	G, Hs	Cu	Fr	Pa		T	0.23	MFK 170 (DACB)

35	*Cassia fistula* L.	Bandar lathi, Gurmata, sonali	Golden shower, Indian laburnum, purging cassia	Caesalpiniaceae	T	Rs	Cu	Fr	P		T	1.63	MFK 149 (DACB)

36	*Cassia occidentalis *L.	Bara kalkesunde	Coffee senna	Caesalpiniaceae	H	Vt, Wp	W	R	Pa	G	O	0.39	JRK 79 (DACB)

37	*Cassia sophera* L.	Kalkeshande	Periwinkle	Caesalpiniaceae	H	Hf, Hs	W	R	Pa	Black pepper	O	0.15	JRK 11 (DACB)

38	*Cassia tora* L.	Chakunda, Panevar	Foetid cassia	Caesalpiniaceae	H	Rs, Wp	W	L, R, S	P		O	0.10	JRK 19 (DACB)

39	*Catharanthus roseus *G. Don	Noyntara	Madagascar periwinkle	Apocynaceae	H	Hf	W	L	Pa	W	T	2.28	MFK 53 (DACB)

40	*Chenopodium album* L.	Betu sak, Betua sak	Fat-hen, goose foot, lamb's quarters	Chenopodiaceae	H	G	Cu	Fr, R	Pa		T	0.61	MSBS 51 (DACB)

41	*Cissampelos pareira * L.	Akanadi, Eklija, Nemuka	False pareira brava	Menispermaceae	Cl	Hf	W	R	D, Pa	Pepper	O	0.28	MSBS 57 (DACB)

42	*Cissus adnata *Roxb.	Bhatia lota	Endeavour river vine	Vitaceae	Wc	Hf	W	L	Pa		T	0.37	MSBS 37 (DACB)

43	*Cissus javana* DC.	Kongngouyen laba	Climbing begonia	Vitaceae	Ch	Hf	W	L, St	Pa	Lw	T	0.12	MSBS 64 (DACB)

44	*Clerodendrum viscosum* Vent.	Bhant	*Clerodendrum *	Verbenaceae	Sh	Hf, Rs, Vt, Wp	W	Fl, L	Pa	W	T	0.23	MFK 207 (DACB)

45	*Clitoria ternatea *L.	Aparajita	Baby watermelon, ivy gourd	Fabaceae	Cl	Hs	Cu	R	P	M	O	0.31	MSBS 114 (DACB)

46	*Costus speciosus* (J. Koenig) Sm.	Kneu	Costus	Costaceae	H	Hf, Rs	W	Bu, L, S	I, J, Pa	W	O, T	3.01	MSBS 62 (DACB)

47	*Curculigo orchioides *Gaertn.	Talmuli	Black musale	Hypoxidaceae	H	F, Hf	W	Bu, L	D, I	M, W	O	0.35	MSBS 94 (DACB)

48	*Cycas pectinata* Griff.			Cycadaceae	T	F	W	Fl	Pa		T	0.49	JRK 18 (DACB)

49	*Cynodon dactylon* Pers.	Durba, Dubla	Bahama grass, Bermuda, Dhub grass	Poaceae	Gr	G, Vt, Wp	W	Wp	Pa		T	0.12	MFK 201 (DACB)

50	*Cyperus rotundus* L.	Bada, Mutha	Nut grass	Cyperaceae	Gr	G	W	Bu	P	Butter	T	0.17	JRK 25 (DACB)

51	*Datura metel * L., *D. fastuosa* L., *D. alba *Nees	Dhutura	Thorn apple	Solanaceae	Sh	Rs, Wp	W	L, S	Pa		T	0.81	JRK 64 (DACB)

52	*Desmodium gangeticum* (L.) DC.	Shalparni	Salpani	Fabaceae	H	F, Hf, Rs	W	Wp	D	M, W	O	2.24	MFK 212 (DACB)

53	*Desmodium triflorum *(L.) DC.	Tripatri		Fabaceae	Us	Mp	W	Sh	J		O	0.72	JRK 12 (DACB)

54	*Eclipta prostrata* L., *E. alba* (L.) Hassk	Kalokesh, Keshori, Keshrangan, Kesuti, Keysuria	False daisy	Asteraceae	H	F, Hf	Cu	L	Pa		T	0.75	MFK 150 (DACB)

55	*Emblica officinalis* Gaertn., *Phyllanthus emblica* L.	Amla, Amalaki, Amluki	Emblic myrobalan, Indian gooseberry	Euphorbiaceae	T	F, Hs	Cu	St	I		O	2.84	MFK 51 (DACB)

56	*Entada rheedii *Spreng.	Gila lata		Mimosaceae	Wc	F, Hf	W	L	Pa		T	0.06	JRK 29 (DACB)

57	*Erythrina variegata* L., *E. indica *Lam.	Mandar, Palita mandar	Coral tree	Fabaceae	T	Hs	Cu	Fl, Rb	Pa		O, T	1.42	JRK 13 (DACB)

58	*Euphorbia hirta* L.	Bara kerui, Baro keruee, dudhia		Euphorbiaceae	H	Vt, Wp	W	R, Wp	J	W	O	0.37	JRK 22 (DACB)

59	*Ficus racemosa* L.	Jagya dumur, Yajna dumur		Moraceae	T	Vt	W	B, S	D, Pa		O, T	0.38	JRK 50 (DACB)

60	*Flacourtia indica* (Burm. f.) Merr.	Benchi		Flacourtiaceae	Sh	F, Hf	W	L	Pa		T	0.26	MSBS 207 (DACB)

61	*Gmelina arborea *L.	Gamar	Indian sarsaparilla	Verbenaceae	T	Hf, Hs	W	R	Pa	H	O	1.37	MSBS 73 (DACB)

62	*Hedyotis scandens *Roxb.	Bijoma		Rubiaceae	Sc	Mp	W	L, St	Pa		T	0.14	MFK 224 (DACB)

63	*Helicteres isora* L.	Atmora	East Indian screw tree	Sterculiaceae	Sh	F, Vt	W	R	D		O	0.19	MSBS 233 (DACB)

64	*Hemidesmus indicus* (L.) R. Br.	Anantamul	Country sarsaparilla, Indian sarsaparilla	Apocynaceae	Cl	Hs, Rs, Vt	W	R	Pa		O	2.75	MFK 218 (DACB)

65	*Holarrhena antidysenterica *(Heyne ex Roth.) Conessi	Kurchi, indrajab		Apocynaceae	T	F, Hf	W	B, R	Pa	W	O, T	0.38	JRK 14 (DACB)

66	*Holarrhena pubescens *(Buch.-Ham.) Wall.	Kurchi		Apocynaceae	T	Hf	W	S	Pa		T	0.51	MFK 217 (DACB)

67	*Homalomena aromatica *(Roxb. ex Sims) Schott.	Gandhabi		Araceae	H	Hf	W	Rh	Pa		T	0.40	JRK 55 (DACB)

68	*Hyptis suaveolens *(L.) Poit.	Bilati tulsi, Tokmadana	Pignut	Lamiaceae	H	Hf, Mp	W	L	J		T	1.50	JRK 16 (DACB)

69	*Ichnocarpus frutescens *(L.) Br.	Dudhilata		Apocynaceae	Cl	Hf, Hs	W	R	Pa		O	2.19	MFK 55 (DACB)

70	*Ixora cuneifolia *Roxb.	Kha muchey		Rubiaceae	Sh	F	W	B	Pa		T	0.03	JRK 20 (DACB)

71	*Jatropha gossypifolia* L.	Lal verenda	Bellyache nettle spurge	Euphorbiaceae	Sh	F, Rs	W	L, S	D	W	O	0.27	MFK 46 (DACB)

72	*Justicia gendarussa *Burm.	Jagatmadan	Indian lilac	Acanthaceae	Sh	Hf, Hs	W	L	Pa		O	2.15	MFK 247 (DACB)

73	*Lantana camara *L.	Kutus kanta		Verbenaceae	Sh	Hf	W	Fl, R, St	D		O	1.46	MSBS 170 (DACB)

74	*Leucas aspera *(Willd.) Link.	Danda kalas	Litchi	Lamiaceae	H	Hf, Hs	W	L	J, Pa	Black pepper, garlic	In	2.67	MFK 33 (DACB)

75	*Mangifera indica* L.	Aam	Mango	Anacardiaceae	T	G, Hs	Cu	L	Pa		T	1.76	MSBS 181 (DACB)

76	*Melastoma malabathricum *L.	Bomang raja	Indian rhododendron	Melastomataceae	Sh	F	W	L	J		T	0.30	MFK 232 (DACB)

77	*Melochia corchorifolia *L.	Bonpat, Tikiokra	Chocolate weed	Sterculiaceae	T	F, Vt	Cu	L	Pa		T	0.18	MSBS 169 (DACB)

78	*Mikania cordata* (Burm. f.) Roxb.	Ashamlata	Climbing hemp	Asteraceae	Sc	Hf, Rs, Wp	W	L	Pa		T	0.31	MFK 199 (DACB)

79	*Mimosa pudica* L.	Lajak, Lajjabati, Lajwati	Sensitive plant	Mimosaceae	Wh	Hs, Rs, Vt	W	L, R	Pa		T	2.11	MFK 94 (DACB)

80	*Mirabilis jalapa* L.	Krishnakali	Marvel of Peru	Nyctaginaceae	H	G, Mp	Cu	L	J	W	O, T	0.16	MSBS 142 (DACB)

81	*Morinda angustifolia* Roxb.	Rang gach		Rubiaceae	Sh	F	W	L	J		O	0.09	JRK 15 (DACB)

82	*Morinda persicifolia* Ham.	Rang gach		Rubiaceae	Sh	F	W	L	Pa		T	2.06	MFK 10 (DACB)

83	*Moringa oleifera *Lamk.	Sajna		Moringaceae	T	Hs	Cu	R	Pa		T	2.45	MFK 16 (DACB)

84	*Mucuna pruriens *Baker	Alkushi	Curry leaf tree	Fabaceae	Cl	Hf, Hs	W	S	Pa		T	1.29	JRK 60 (DACB)

85	*Mussaenda roxburghii *Hook. f.	Ranirtak		Rubiaceae	Sh	F, Mp	W	L	Pa		T	0.27	JRK 41 (DACB)

86	*Ocimum basilicum *L.	Babul tulsi, Kali tulsi, Pashanbeddie	Common basil, sweet basil	Lamiaceae	H	F, Rs	W	Wp	Pa		T	0.96	MSBS 98 (DACB)

87	*Oroxylum indicum* Vent.	Nasona, Sona, Sonpatti	Broken bones, Indian trumpet flower, midnight horror	Bignoniaceae	T	F, Hf, Vt	W	B	Pa		T	2.02	MFK 175 (DACB)

88	*Oxalis corniculata* L.	Amrul shak, Chuka tripati	Indian sorrel, yellow oxalis	Oxalidaceae	H	Mp	W	L	Pa		T	0.86	MSBS 32 (DACB)

89	*Peliosanthes teta *Andr.	Fuji ghash		Liliaceae	H	F	W	Rt	Pa		T	0.36	MSBS 53 (DACB)

90	*Peperomia pellucida* (L.) HBK	Luchi pata	Peperomia, shiny bush	Piperaceae	H	Vt	W	Sh	J		O	0.15	JRK 21 (DACB)

91	*Piper nigrum* L.	Golmorich, Kala morich	Black pepper	Piperaceae	Cl	G	Cu	R	Pa		T	0.57	MFK 181 (DACB)

92	*Plumbago zeylanica* L.	Chita, Chitrak, Sada chita, Sufaid	Ceylon leadwort, white flowered leadwort	Plumbaginaceae	H/Us	F, G	Cu, W	R	Pa		T	0.28	JRK 155 (DACB)

93	*Polygonum chinense *L.		Chinese knotweed	Polygonaceae	H	Rs, Vt	W	L	Pa		T	0.15	JRK 2133 (DACB)

94	*Pouzolzia indica *Gaud.	Bormajal		Urticaceae	H	Mp	W	L	Pa		T	0.16	MFK 69 (DACB)

95	*Premna esculenta *Roxb.	Lelom pata		Verbenaceae	S	F	W	L	Pa		T	0.06	MFK 77 (DACB)

96	*Rauvolfia serpentina *(L.) Benth. ex Kurz	Sarpagandha	Snakeroot	Apocynaceae	H	Hf, Hs	Cu, W	L, R, Rh	D, Pa	M	O, T	3.98	MFK 66 (DACB)

97	*Senna hirsuta *(L.) Irwin & Barneby	Kanduak	Foetid senna	Caesalpiniaceae	H	F, Mp	W	L	Pa		T	0.73	MSBS 17 (DACB)

98	*Senna tora* (L.) Roxb.	Chakunda, Kalkasham	Metal seed, sickle senna	Caesalpiniaceae	H	Rs, Wp	W	L	Pa		T	0.14	MSBS 144 (DACB)

99	*Sida acuta *Burm.	Kureta	Country mallow	Malvaceae	Wh	Hf, Hs	W	L	Pa		T	1.98	MFK 61 (DACB)

100	*Sida cordifolia* L.	Bala, Barela	Country mallow	Malvaceae	H	F	W	L	J		O	0.10	MSBS 191 (DACB)

101	*Sida rhombifolia* L.	Kureta, Lal berela	Cuba jute, Queensland hemp	Malvaceae	H	Hf, Wp	W	L, St	J, Pa		O, T	0.25	JRK 170 (DACB)

102	*Solanum torvum *Swartz.	Gota, Titbegun		Solanaceae	Sh	Hf, Hs	W	R	J	Mustard oil, ammonium chloride	O, T	0.31	JRK 112 (DACB)

103	*Streblus asper* Lour.	Sheowra	Siamese rough bush	Moraceae	T	Hf	W	R	J, P		T	0.21	MFK 117 (DACB)

104	*Syzygium cumini *(L.) Skeels., *Eugenia jambolana* Lamk.	Jam, Kalojam	Black plum, Indian black berry	Myrtaceae	T	G, Hs	Cu,	B	Pa		T	1.93	MFK 130 (DACB)

105	*Tamarindus indica *L.	Tetai, Tetul, Tintil	Tamarind tree	Caesalpiniaceae	T	G, Hs	Cu	Wp	P	H	O	1.68	JRK 57 (DACB)

106	*Tephrosia purpurea *(L.) Pers.	Ban nil	Fish poison, wild indigo	Fabaceae	Sh	Rs	Cu, W	R	Pa		T	0.07	JRK 30 (DACB)

107	*Terminalia arjuna* (Roxb.) Wt. & Arn.	Arjun, Arjuna, Kahu		Combretaceae	T	F	Cu	Stb	P, Pa	H, M/black pepper	O	1.89	MSBS 76 (DACB)

108	*Tinospora cordifolia *(Willd.) Miers	Gulancha, Gurach	Heart leaved moon seed	Menispermaceae	Cl	F, Rs	W	St	J		O	0.22	MSBS 18 (DACB)

109	*Trewia nudiflora *L.	Bhatam, Betul, Pitali	False white teak	Euphorbiaceae	T	F, Mp	W	L	Pa		T	0.18	MFK 114 (DACB)

110	*Trichosanthes tricuspidata *Lour.	Makal		Cucurbitaceae	Ch	F	W	R	J		O	0.09	MFK 162 (DACB)

111	*Tylophora indica* (Burm. f.) Merrill.	Abtomul		Asclepiadaceae	H	F, Vt	W	L	Pa	Urine	In	0.21	JRK 100 (DACB)

112	*Urena lobata* L.	Ban okra		Malvaceae	H	F	W	R	D		O	0.14	MSBS 06 (DACB)

113	*Vitex negundo* L.	Nirgundi, Nishinda, Sundubar	Indian privet	Verbenaceae	Sh/T	F, Hf	W	L, R, Rh	Pa		T	2.58	MFK 100 (DACB)

114	*Vitis lanata *Roxb.			Vitaceae	Sc	F, Hf	Cu, W	L	Pa		T	0.06	MSBS 50 (DACB)

115	*Willughbeia edulis *Roxb.			Apocynaceae	Cl	F	W	St	Pa		T	0.08	JRK 75 (DACB)

116	*Withania somnifera *Dunal	Ashwagandha	Spanish bayonet, Spanish dagger	Solanaceae	H	Hs	Cu	R	Pa		T	1.25	JRK 140 (DACB)

THP = traditional health practitioners; habit: Sc = shrubby climber, Wh = woody herb, C = climber, Cr = creeper, Sh = shrub, H = herb, and T = tree; habitat: Hf = hill forest, Hs = homestead, Wp = waste place, Rs = roadside, Vt = village thicket, G = garden, F = forest, and Mp = marshy place; nature: Cu = cultivated, W = wild; plants parts used: B = bark; Bu = bulb, L = leaves, Fr = fruit, Fl = flower, Gp = ground plant, Pe = petioles, R = root, Rh = rhizome, S = seeds, S = shoots, St = stem, Stb = stem bark, Tr = tuberous root, Sy = styles, and Wp = whole plant; preparation: D = decoction, J = juice, I = infusion, C = cook, R = raw, P = powder, and Pa = paste; solvent/adjuvant used: M = milk, H = honey, W = water, and Wi = wine; mode of application: O = oral, T = topical, and V = vaginal; relative abundance: C = common, Lc = least common, and R = rare; conservation status: LC = least common, VU = vulnerable, NT = near threat, UV = use value, and FC = frequency of citation. CITES = endangered commercial plant species; WHO = World Health Organization; FL = fidelity level; Fic = informant consensus factor; RI = relative importance value; FL = fidelity level; ICD = International Statistical Classification of Diseases and Related Health Problems.

**Table 3:** Doses of the available plants.

S/L number	Name of the plants	Doses
1	*Abelmoschus moschatus *Medic.	Paste of leaf, fruit, and seed is used on the infected area 2/3 times daily for 2/3 days. Juice of leaf, fruit, and seed is also taken by grinding with milk and sugar.

2	*Abroma augusta* Linn. f.	Root juice is used after maceration.

3	*Abrus precatorius *L.	Seed powder is mixed with *Andrographis paniculata* seed powder to consume with lemon juice.

4	*Acacia farnesiana* (L.) Willd.	3-4 pieces of fresh root are crushed and squeezed; the extract is taken 3–5 times a day for 1 day.

5	*Acalypha indica* L.	Whole plant is made into paste, and the paste thus obtained is divided into 4-5 equal parts; each part is given at 6-hour intervals as an antidote.

6	*Achyranthes aspera* L.	Fresh leaves extract of about 2 teaspoonfuls is given 4–6 times a day.

7	*Acorus calamus* L.	Fresh rhizome is made into paste. The paste is given with a glass of lukewarm water twice a day for 3 successive days.

8	*Aegle marmelos* (L.) Corr. Serr.	Whole plants, in tender condition, are made into paste. The paste thus obtained is divided into two equal halves, one half mixed with rice beer applied for cleaning the biting site and the other half again divided into 6 equal portions, and each part is given at an interval of 4–6 hours of time for one day.

9	*Albizia procera* (Roxb.) Benth.	Fresh root of about one inch long, collected from the plants which are yet not flowered, is given once as an antidote of snakebite.

10	*Allium cepa* L.	Two teaspoonfuls of bulb juice of the plant mixed with mustard oil and administered to expel poison by vomiting.

11	*Andrographis paniculata *(Burm. f.) Wall. ex Nees	(1) Fresh root and leaves are mixed in a ratio of 2 : 3 and the whole mixture is made into paste with a little water. The paste thus obtained is divided into 12–16 equal parts (based on the condition of the patient) and each part is given at regular intervals of 1-2 hours for 2-3 days. (2) Root paste along with honey in equal parts is given 6–8 times a day as an antidote of snake venom.

12	*Annona squamosa* L.	Incision of snakebite is washed with the juice of plants.

13	*Aristolochia indica* L.	(1) Fresh root extract mixed with equal amount of root extract of *Rauvolfia serpentina* is given 4–6 times a day. (2) Root paste along with honey in equal parts is given 6–8 times a day.

14	*Asparagus racemosus *Willd.	Leaf extract is applied on the bitten area.

15	*Bacopa monnieri* (L.) Pennell	Dried plant (except the root portion) powder, about 1 teaspoonful, is given with a cup of warm goat milk or black tea 2-3 times of day as an antidote of snakebite.

16	*Baliospermum montanum *L.	Paste is prepared with leaf and applied externally twice a day for 4/5 days.

17	*Begonia barbata *C.B. Clarke	Paste is prepared with stem and leaves and applied once a day for 2/3 days.

18	*Bombax ceiba* L.	Fresh young shoots (3–5 pieces) are made into paste with black peppers seed (*Piper nigrum*) and a pinch of camphor (Karpur); the paste thus obtained is given after mixing with a spoonful of honey as an antidote of snake venom.

19	*Butea monosperma* (Lamk.) Taub.	Stem bark (fresh or dried) about 20 g is made into paste with zinger (rhizome of *Zingiber officinale*). The whole paste thus obtained is divided into four equal parts. Each of these 4 parts is given 4 times a day.

20	*Byttneria pilosa *Roxb.	Paste of stem and leaf is applied twice a day until the area is cured.

21	*Cajanus cajan *(L.) Huth.	Paste is made with seed powder of the plant and the juice of leaf of *Senna tora*. It is then applied twice a day for 2/3 days.

22	*Calotropis gigantea* (L.) Ait. f.	Fresh root with milk of cow is ground to a fine paste and taken as an antidote for snakebite.

23	*Calotropis procera *(Ait.) Ait. f.	About three drops of latex are put on the snake-bitten area and pressed downwards to bleed; root extract is given two cups a day; flower powder is mixed with black pepper and taken.

24	*Calycopteris floribunda* Lamk.	Root juice is used in infected area.

25	*Capparis zeylanica* L.	Dried fruits with seeds are made into dust; this dust is given as 1 teaspoonful with a glass of lukewarm water as a snake venom antidote.

26	*Cassia fistula* L.	As a remedy against snakebite, one teaspoonful fruit powder is taken internally.

27	*Cassia occidentalis *L.	20–30 gm of root (fresh or dried) is made into paste with 3-4 pieces of “garlic” (*Allium sativum*) and a little “gur” (Jaggery); the whole mixture thus obtained is given as an antidote to snakebite.

28	*Cassia sophera* L.	Root (fresh or dried) of about 20 gm is made into paste with 5–7 pieces of black peepers (seeds of *Piper nigrum*) and the paste is given as an antidote.

29	*Catharanthus roseus D. Don *	Leaf is grinded after maceration.

30	*Cissampelos pareira * L.	Root paste with 10 g long pepper is prescribed once daily for 5 days.

31	*Cissus adnata *Roxb.	Leaf paste is applied on infected place.

32	*Cissus javana* DC.	Paste is made with leaf and stem, mixing with lime, and applied externally on the biting place tying a piece of cloth for 3/4 days.

33	*Clitoria ternatea *L.	Root powder mixed with milk is taken orally immediately after snakebite.

34	*Cycas pectinata *Griff.	Paste of flower is applied thrice a day for 2/3 days.

35	*Cyperus rotundus* L.	Bulb powder mixed with cow butter to treat snakebite.

36	*Desmodium triflorum *(L.) DC.	Juice is prepared with shoots and mixing with shoots of *Peperomia pellucida* and 2 spoonfuls are taken thrice a day for 4/5 days.

37	*Emblica officinalis* Gaertn.	Stem infusion is given orally as an antidote.

38	*Entada rheedii *Spreng.	Paste is prepared with leaf and applied externally once a day for 4/5 days.

39	*Erythrina variegata* L.	Stamen and root bark are mixed in a ratio of 1 : 3 and then they are made into paste. This paste is applied in both ways externally and internally to reduce the swelling, pain of snakebite.

40	*Ficus racemosa* L.	A few drops of its decoction are put into the nostrils, resulting into vomiting and relief; bark paste is applied over the injury.

41	*Gmelina arborea *L.	Inner portion of fresh root (after peeling off the bark) about 20 g is made into paste and this paste is given with a spoonful of honey as an antidote of snakebite.

42	*Hedyotis scandens *Roxb.	Paste is prepared with leaf and stem and applied in warmed condition externally twice a day for 3/4 days.

43	*Hemidesmus indicus* (L.) R. Br.	Root paste is applied as an antidote to snakebite.

44	*Holarrhena antidysenterica *(Heyne ex Roth.) Conessi	The roots were rubbed on a stone with a few drops of water and the paste obtained is given internally and applied externally in snakebite.

45	*Holarrhena pubescens *(Bach.-Ham.) Wall.	Seed paste is applied locally as antidote and also for reducing the swelling and pain of snakebite.

46	*Homalomena aromatica *(Roxb. ex Sims) Schott.	Paste of rhizomes is applied until the area is cured.

47	*Hyptis suaveolens *(L.) Poit.	Juice is extracted from leaf and applied externally twice a day for 3 days.

48	*Ichnocarpus frutescens *(L.) Br.	Fresh roots (about 100 g) are crushed and squeezed; the aqueous extract thus obtained is given 10–12 times a day as an antidote.

49	*Ixora cuneifolia *Roxb.	Bark is grinded with water and the paste applied on the biting area twice a day for 4/5 days.

50	*Justicia gendarussa *Burm.	Fresh leaves extract is given 20–30 mL at every 1-hour interval for up to 18 hours of snakebite as an antidote.

51	*Lantana camara *L.	Decoction of roots, flowers, and stems is prescribed.

52	*Leucas aspera *(Willd.) Link.	Leaves with pepper and garlic are chewed and spit into the nostrils.

53	*Melastoma malabathricum *L.	Juice is prepared with leaf and applied externally twice a day for 3/4 days.

54	*Melochia corchorifolia *L.	Leaf paste is applied on infected place.

55	*Mirabilis jalapa *L.	Leaf juice is extracted and 2 spoonfuls are taken twice a day for 2/3 days. Also it is applied topically twice a day in infected areas.

56	*Morinda angustifolia* Roxb.	4 spoonfuls of extracted leaf juice are taken thrice a day until the area is cured.

57	*Morinda persicifolia* Ham.	Paste is prepared with leaf and applied externally twice a day for 3/4 days.

58	*Mussaenda roxburghii *Hook. f.	Paste of leaf is applied on the infected place with tying a piece of cloth.

59	*Peliosanthes teta *Andr.	Paste is prepared with root tuber and applied externally once a day for 2 days.

60	*Peperomia pellucida *(L.) HBK	Juice is prepared with shoots and mixing with shoots of *Desmodium triflorum* and 2 spoonfuls are taken thrice a day for 4/5 days.

61	*Polygonum chinense *L.	Paste is prepared with leaf and applied externally once a day for 2 days.

62	*Pouzolzia indica *Gaud.	Paste of leaves is applied twice a day for 2/3 days.

63	*Premna esculenta *Roxb.	Paste is prepared with leaf and applied externally twice a day for 3/4 days.

64	*Rauvolfia serpentina *(L.) Benth. ex Kurz	Roots and leaf buds are crushed with milk and made into a paste and used internally and externally on the affected area; rhizome and root decoction is given orally.

65	*Senna hirsuta *(L.) Irwin & Barneby	Paste of leaf is applied topically on the biting place.

66	*Senna tora *(L.) Roxb.	Paste is prepared with leaf and applied once a day for 2/3 days.

67	*Sida cordifolia* L.	Leaf juice is applied to cure snakebite.

68	*Sida rhombifolia *L.	Paste is prepared with leaf and stem and applied topically once a day to infected areas. Also juice of extracted leaf and stem is 2 spoonfuls which are taken four times a day for 4/5 days.

69	*Solanum torvum *Swartz.	Root juice is mixed with 250 mL water and 100 mL mustard oil. First, ammonium chloride is rubbed on the snake-bitten area and then the mixture of root juice, water, and oil is given orally. Otherwise, 1 handful of fruit is boiled in 1/2 litre of water. The fruits are then squeezed to get the juice, which is orally given to the snake-bitten person to vomit out the poison.

70	*Tamarindus indica *L.	To treat snakebite, spoonful powder with honey is consumed thrice a day after every two hours.

71	*Terminalia arjuna* (Roxb.) Wt. & Arn.	Stem bark powder (about 10 g) is made into paste with a teaspoon full of honey and 5–7 pieces of black pepper (*Piper nigrum*); this paste is given with a glass of lukewarm goat milk as an antidote to snake venom.

72	*Tinospora cordifolia *(Willd.) Miers	Stem juice is used to cure snakebite.

73	*Trewia nudiflora *L.	Paste of leaf is applied topically on the biting place.

74	*Trichosanthes tricuspidata *Lour.	Root juice is prepared after maceration and 1 spoonful is taken twice a day until the area is cured.

75	*Tylophora indica* (Burm. f.) Merrill.	Handful of leaves is crushed in urine of snake-bitten person and 2-3 drops of extract are passed through the nostrils.

76	*Urena lobata* L.	Decoction of root along with leaves of *Adhatoda vasica, Alangium salvifolium,* and *Cocciniagrandis* is taken internally.

77	*Vitis lanata *Roxb.	Paste is prepared with leaf and applied externally once a day for 3/4 days.

78	*Willughbeia edulis *Roxb.	Latex is collected from stem and applied externally thrice a day for 2/3 days.

**Table 4:** Worldwide comparative studies of cited plants of our survey.

Serial number	Scientific name of the plant	Region/country	Reference(s)
1	*Abelmoschus moschatus *Medic.	West Bengal, India	[39, 40]
2	*Abrus precatorius *L.	Arunachal Pradesh, India	[41]
3	*Acacia farnesiana* (L.) Willd.	West Bengal, India; Brazil	[42, 43]
4	*Acalypha indica* L.	West Bengal, India	[44]
5	*Achyranthes aspera* L.	West Bengal, India	[39, 43, 44]
6	*Ageratum conyzoides* L.	Meghalaya, India	[45]
7	*Albizia lebbeck* (L.) Benth.	Uttar Pradesh, India; Islamabad, Lahore, Pakistan	[46–48]
8	*Albizia procera (*Roxb.) Benth.	West Bengal, India	[49]
9	*Allium cepa* L.	Rajasthan, India; Brazil; Kenya	[33, 43, 50]
10	*Andrographis paniculata *(Burm. f.) Wall. ex Nees	West Bengal, India	[44]
11	*Annona squamosa* L.	Nicobar, India	[51]
12	*Argemone mexicana* L.	Rajasthan, India; Brazil	[43, 52]
13	*Aristolochia indica* L.	Karnataka, Madhya Pradesh, Orissa, Tamil Nadu, West Bengal, India	[53–57]
14	*Asparagus racemosus *Willd.	Karnataka, India	[58]
15	*Bacopa monnieri *(L.) Pennell	West Bengal, India	[59]
16	*Bauhinia variegata* L.	Rupandehi District, Nepal; Islamabad, Pakistan	[47, 60, 61]
17	*Bixa orellana* L.	West Bengal, India	[59]
18	*Bombax ceiba* L.	West Bengal, India	[49]
19	*Buchanania lanzan* Spreng.	Tamil Nadu, Uttar Pradesh, India	[48, 62]
20	*Butea monosperma* (Lamk.) Taub.	West Bengal, India	[49]
21	*Calotropis procera (*Ait.) Ait. f.	Rajasthan, Tamil Nadu, India; Balochistan, Pakistan	[57, 63, 64]
22	*Calotropis gigantea* (L.) Ait. f.	Orissa, India	[65]
23	*Capparis zeylanica* L.	West Bengal, India	[44]
24	*Capsicum annuum* L.	India	[66]
25	*Cassia fistula* L.	Karnataka, Tamil Nadu, Uttar Pradesh, India	[48, 54, 62]
26	*Cassia occidentalis* L.	West Bengal, India	[59]
27	*Cassia sophera* L.	West Bengal, India	[39]
28	*Cassia tora* L.	Uttaranchal, Uttar Pradesh, India; Brazil	[43, 48, 67]
29	*Chenopodium album* L.	Islamabad, Pakistan	[47]
30	*Cissampelos pareira * L.	Rajasthan, West Bengal, Tamil Nadu, India; Islamabad, Pakistan	[47, 55, 68, 69]
31	*Clitoria ternatea *L.	Madhya Pradesh, Meghalaya, Uttar Pradesh, India	[48, 70, 71]
32	*Cynodon dactylon* (L.) Pers.	India	[72]
33	*Cyperus rotundus* L.	Madhya Pradesh, India	[71]
34	*Datura metel *L.	Nicobar, Uttar Pradesh, India	[48, 51]
35	*Eclipta prostrata * L.	India; Brazil	[43, 72]
36	*Emblica officinalis *Gaertn.	Maharashtra, India	[73]
37	*Ficus racemosa* L.	Rajasthan, India	[74]
38	*Flacourtia indica *(Burm. f.) Merr.	Tamil Nadu, India	[62]
39	*Gmelina arborea* L.	West Bengal, India	[49]
40	*Helicteres isora *L.	Uttar Pradesh, India	[48]
41	*Hemidesmus indicus *(L.) R. Br.	West Bengal, India; Sri Lanka	[55, 75]
42	*Holarrhena antidysenterica *(Heyne ex Roth.) Conessi	Orissa, India	[65]
43	*Holarrhena pubescens* (Bach.-Ham.) Wall.	West Bengal, India	[44, 49]
44	*Hyptis suaveolens *(L.) Poit.	Uttar Pradesh, India	[48]
45	*Ichnocarpus frutescens* (L.) Br.	West Bengal, India	[44]
46	*Lantana camara* L.	Madhya Pradesh, India	[71]
47	*Mangifera indica* L.	Uttar Pradesh, India	[76]
48	*Mimosa pudica* L.	Nagaland, Uttar Pradesh, India	[48, 77]
49	*Moringa oleifera* Lamk.	Assam, India	[78]
50	*Mucuna pruriens *Baker	Uttar Pradesh, India	[48]
51	*Ocimum basilicum* L.	Uttar Pradesh, India; Brazil	[43, 48]
52	*Oroxylum indicum *Vent.	Orissa, India	[79]
53	*Oxalis corniculata* L.	Meghalaya, India; Tehsil Chakwal, Pakistan	[45, 80]
54	*Piper nigrum* L.	Uttaranchal, India	[81]
55	*Plumbago zeylanica* L.	Tripura, India	[82]
56	*Rauvolfia serpentina *(L.) Benth. ex Kurz	Karnataka, Tamil Nadu, Uttar Pradesh, India	[48, 83, 84]
57	*Sida acuta *Burm.	Madhya Pradesh, India	[71]
58	*Sida cordifolia *L.	Rajasthan, Tamil Nadu, India	[52, 84]
59	*Syzygium cumini* (L.) Skeels.	Orissa, India	[85]
60	*Tamarindus indica* L.	Maharashtra, India; Africa; Sudan	[75, 86]
61	*Terminalia arjuna* (Roxb.) Wt. & Arn.	West Bengal, India	[49]
62	*Tinospora cordifolia* (Willd.) Miers	Madhya Pradesh, Tamil Nadu, Uttar Pradesh, India	[62, 76, 87]
63	*Tylophora indica* (Burm. f.) Merrill.	Karnataka, India	[54]
64	*Urena lobata* L.	Tamil Nadu, India	[88]
65	*Vitex negundo* L.	Himachal Pradesh, Kerala, India	[89, 90]
66	*Withania somnifera* (L.) Dunal	Karnataka, India; Pakistan	[58, 75]

**Table 5:** Literature study of the plants surveyed having toxicity.

Scientific name	Toxic part	Toxic compound	Toxic effect	Reference
*Abrus precatorius* L.	Seed	Abrin, ricin	Abortifacient, inhibiting cell protein synthesis	[91]

*Acorus calamus* L.	Seed	Beta-asarone	Procarcinogenic	[92, 93]

*Ageratum conyzoides* L.	Seed	Pyrrolizidine alkaloids lycopsamine, echinatine	Liver lesions and tumors	[94–96]

*Annona squamosa* L.	Root, seed	Annonastin, squamozin	Roots are drastic purgative and seeds are strong eye irritant, abortifacient	[97]

*Argemone mexicana* L.	Seed, latex	Sanguinarine, dihydrosanguinarine	Epidemic dropsy	[98–101]

*Bacopa monnieri* (L.) Pennell	Whole plant		Suppress spermatogenesis and fertility, digestive problem	[102, 103]

*Calotropis gigantea *(L.) Ait. f.	Root	Calotropin	Inhibit spermatogenesis, abortifacient	[104]

*Calotropis procera *(Ait.) Ait. f.	Root	Cytotoxin, calotropin, calcilin, gigantin	Ocular toxicity	[105]

*Cassia occidentalis* L.	Pods and beans	Pyrrolizidine alkaloid	Hepatotoxic	[106–108]

*Catharanthus roseus* (L.) G. Don.	Root, shoot	Vincristine, vinblastine	Hypotension, neurotoxicity, anaemia, seizure	[109]

*Ficus racemosa* L.	Bark	Tetracyclic triterpene derivatives	Cause abnormality of liver and kidney	[110]

*Lantana camara *	Leaf	Triterpene acids	Leaf extracts are cytotoxic	[111]
